# Supplementary figures and images for: Gap junctions in the alimentary tract regulate reproductive span in C. elegans
Source: bioRxiv. 2026 Jun 7:2026.06.03.729750. Preprint. [Version 1] doi: 10.64898/2026.06.03.729750 (PMC13252183; doi:10.64898/2026.06.03.729750)

Figure S1

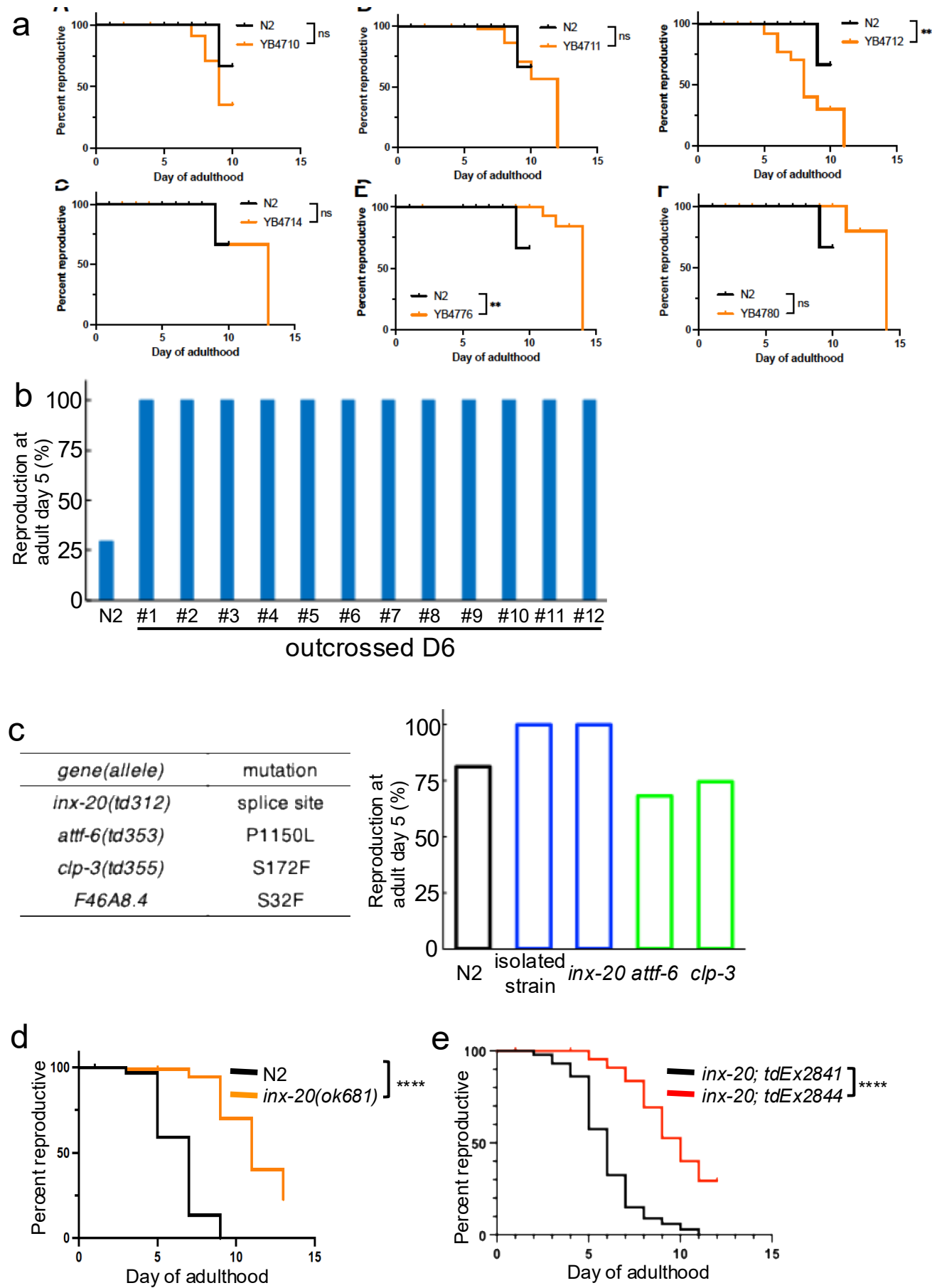

Figure S2

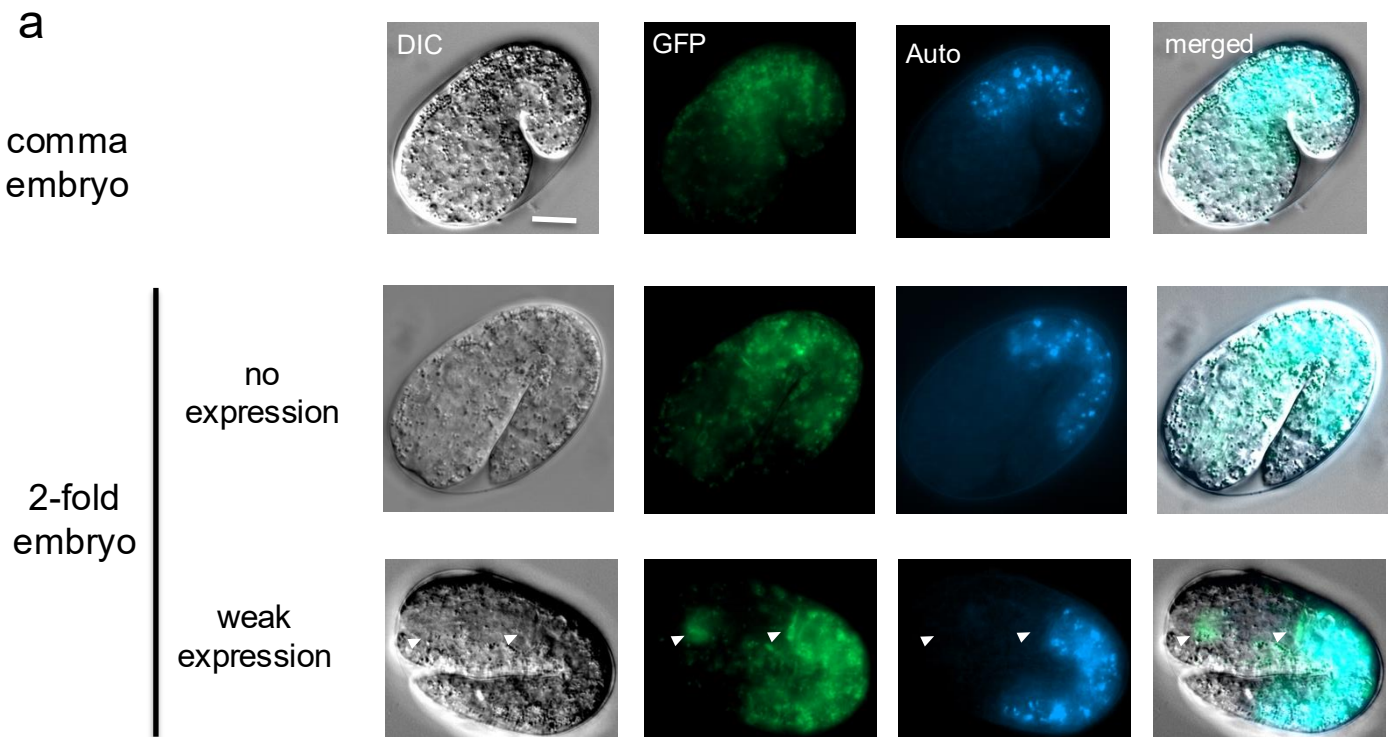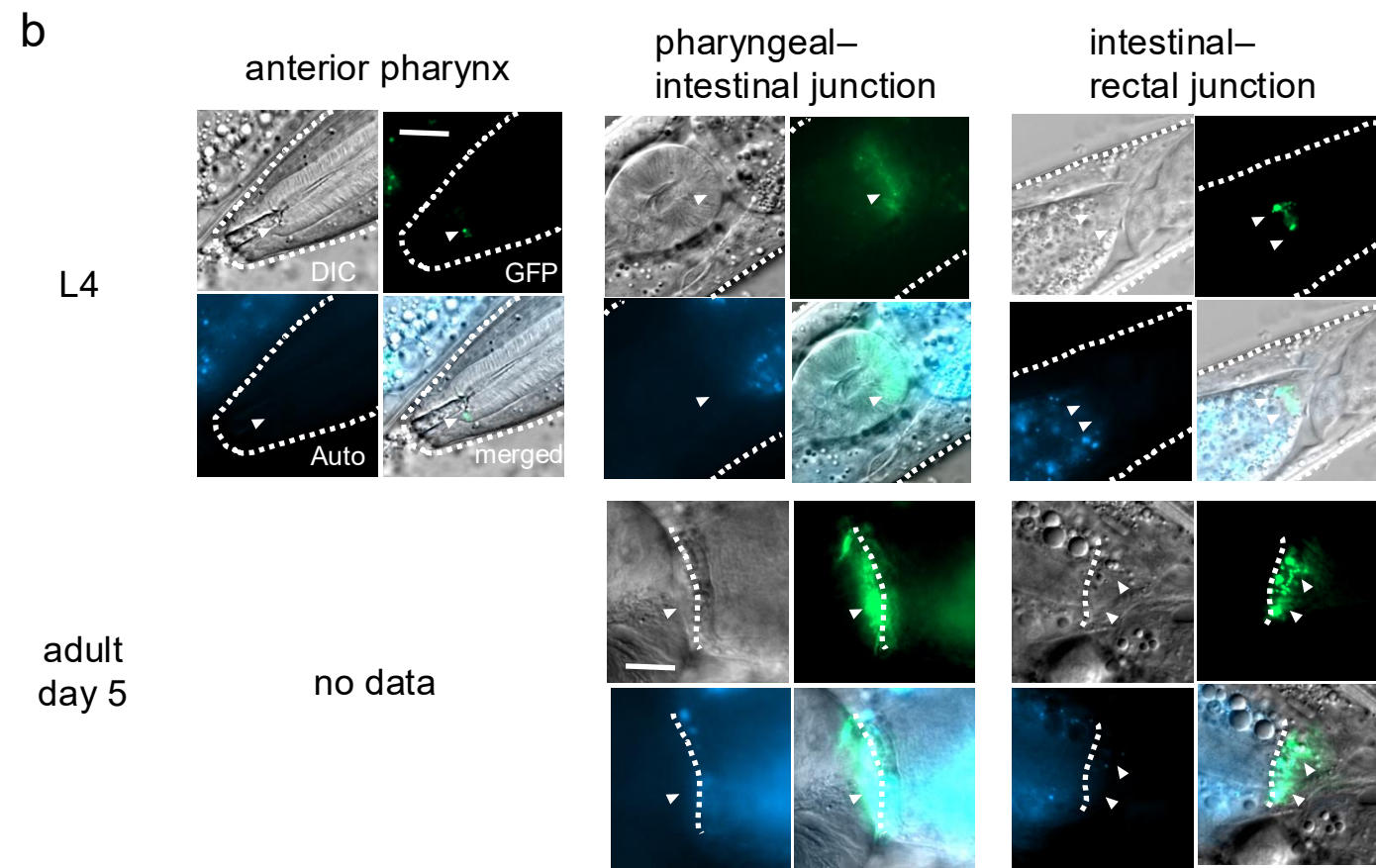

Supplement: Supplement 1 — Figure S1 Isolation and validation of an inx-20 mutant with extended reproductive span (a) Two out of the six strains that exhibit an extended reproductive span under self-fertilization also exhibit an extended reproductive span when mated with N2 males. (b) One strain exhibited increased late reproduction after being outcrossed twice. (c) The isolated strain carried four mutations, and among the four mutated genes, inx-20 was identified as the gene responsible for increased late reproduction. (d) inx-20 loss-of-function mutants exhibit an extended reproductive span under selffertilizing conditions. (e) The extended reproductive span phenotype was rescued by expression of wildtype inx-20. Transgenic animals carrying the extrachromosomal array were identified using Pmyo-3::mCherry::unc-54 3′UTR as a co-injection marker. (a),(d) and (e) Log-rank (Mantel-Cox) test, 30-50 worms per strain, p-value * < 0.05, ** < 0.01, *** < 0.001, **** < 0.0001. Figure S2 Continuous expression of inx-20 from embryogenesis to adulthood (a) INX-20::mSG expression is not detectable at the comma stage. (b) INX-20::mSG expression is maintained from embryogenesis through adult day five. (a) and (b) inx-20 expression is shown in green (panels labeled GFP), and intestinal autofluorescence is shown in blue (panels labeled Auto). Arrowheads indicate inx-20 expression, and the dotted line marks the intestinal boundary. In all panels except the two-fold embryo, anterior is to the left and posterior to the right. At least 10 worms were examined for each condition, and representative images are shown. Scale bar, 10 μm. [file media-1.pdf]
